# Care of Young Invalids

**Published:** 1920-05-08

**Authors:** 


**THE PUBLIC WELL-BEING—(*continued*).**

### Care of Young Invalids.

Good work for invalid children, though on different lines from those of the Care of Cripples Committee, is done by the Invalid Children's Aid Association, whose annual meeting was held last week. It undertakes the care of invalid and crippled children after they come out of hospital, and does admirable work, in association with the London County Council, for the care of pre-tubercular cases. Help of this kind was given to over ten thousand children during the year. It was stated at the meeting, held in Londonderry House, that the Association has seven special convalescent homes, which combine open-air treatment with education, and an eighth can be opened if the necessary funds are forthcoming. Each child costs 30s. a week to keep at the homes, and of this amount at least 12s. has to be raised by the Association. The Association has been recently given the use of a large house, rent free, to be devoted to the treatment of children suffering from tuberculous disease, and £1,000 is required immediately for its equipment. In addition to the ordinary work of the Association, a large expenditure is necessary in connection with the East End Branch. Last year the subscriptions amounted to £1,221, and the donations to £9,407, while the expenditure reached £23,376.

Beneficent work of this character is carried on in many parts of the country in some form or other. There has been in Leeds for the last thirty-four years a Convalescent and Summer Holiday Fund—its annual meeting has just been held—the main purpose of which has been to find country homes for children whose parents are unable to provide a holiday for them. Convalescent patients also from various public institutions are sent to seaside and other places for periods of a few weeks to a few months, according to the particular needs, and a third activity of the fund is the care of mothers and babies by arrangement with the Health Committee. But ordinary work is the provision of holidays. Last year this was done for 200 children, and altogether 600 children were sent away for holiday or for recuperation, at a cost per child of about thirty shillings; the great majority were away for periods under three weeks. This organisation works in friendly co-operation with another Leeds Association, which runs a holiday camp for poor children. The camp is shortly to be kept open all the year round. The pity of it is that so many of these excellent organisations have a struggle to make ends meet.

